# Pleural staging using local anesthetic thoracoscopy in dry pleural dissemination and minimal pleural effusion

**DOI:** 10.1111/1759-7714.13894

**Published:** 2021-02-25

**Authors:** Tatsuya Imabayashi, Yuji Matsumoto, Midori Tanaka, Toshiyuki Nakai, Takaaki Tsuchida

**Affiliations:** ^1^ Department of Endoscopy, Respiratory Endoscopy Division National Cancer Center Hospital Tokyo Japan; ^2^ Department of Thoracic Oncology National Cancer Center Hospital Tokyo Japan

**Keywords:** Dry pleural dissemination, interventional pulmonology, local anesthetic thoracoscopy, minimal pleural effusion, non‐small cell lung cancer

## Abstract

**Background:**

Dry pleural dissemination (DPD) and minimal (<10 mm thick) pleural effusion (PE) may be discovered intraoperatively as unexpected metastases. A definitive diagnostic procedure such as pleural biopsy is rarely attempted in such patients preoperatively. We retrospectively investigated the use and safety of local anesthetic thoracoscopy (LAT) as a pleural staging tool in the diagnosis of DPD and minimal PE.

**Methods:**

We reviewed 18 patients with non‐small cell lung cancer (radiological DPD and minimal PE in 13 and five patients, respectively) who underwent LAT using a flex‐rigid pleuroscope for pleural staging from April 2015 to September 2020.

**Results:**

The median age of the patients was 72 years. Nine patients (50%) were men. The dominant histological type was adenocarcinoma (*n* = 16). Three patients each with radiological DPD and minimal PE had visible PE on the LAT. Pleural biopsy was performed in the 16 cases in which pleural abnormalities were identified. On pleural staging, five cases were diagnosed without pleural dissemination (M0), and 13 cases were diagnosed with pleural dissemination (M1a). Only one case in which the lesion could not be identified because of pleural adhesions was false‐negative. The success rates for pleural staging, sensitivity, specificity, positive predictive value, and negative predictive value were 94.4% (17/18), 92.8% (13/14), 100% (4/4), 100% (13/13), and 80.0% (4/5), respectively. There were no lung lacerations or other severe complications caused by the procedure or during blunt dissection.

**Conclusion:**

LAT might be a useful tool for accurate pleural staging in cases with DPD and minimal PE suspected radiologically.

## Graphical abstract

Local anesthetic thoracoscopy using a single‐puncture technique with a flex‐rigid pleuroscope might be a useful tool for accurate pleural staging in cases wherein dry pleural dissemination and minimal pleural effusion were suspected radiologically.

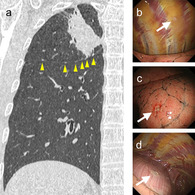

## INTRODUCTION

Non‐small cell lung cancer (NSCLC) patients with pleural metastases, which include malignant pleural effusion (PE) and/or malignant pleural nodules, have poor prognosis.^1^ Pleural metastases of NSCLC, regardless of the extent of PE and pleural nodules, confer an M1a descriptor (stage IVA) in the latest (8th) edition of the tumor‐node‐metastasis (TNM) classification and are usually not indicated for surgical resection.^2^

Computed tomographic (CT) scans have been firmly established in the diagnostic pathway for investigating PE and pleural nodules. Additionally, thoracentesis is often able to diagnose PE and is an appropriate first step.^3^ However, approximately one in three patients with PE is undiagnosed after a single thoracentesis.^4^ The British Thoracic Society guidelines suggest that if the analysis of PE (cytology, protein, lactate dehydrogenase, pH, Gram stain, and culture) does not reveal a cause, then undiagnosed exudative effusions should be further investigated with contrast‐enhanced CT scans, and only then should pleural biopsy, such as radiologically guided biopsy, local anesthetic thoracoscopy (LAT), or video‐assisted thoracoscopic surgery, be considered.^3^

Conversely, in cases of dry pleural dissemination (DPD), defined as solid pleural metastases without PE^5^, ^6^, ^7^, ^8^, ^9^ or minimal PE (<10 mm thick) on CT scans,^10^ which are considered early stages of pleural metastases, it is difficult to radiologically differentiate between benign and malignant lesions, and thoracentesis is not recommended.^11^ Most of these cases are clinically diagnosed as M0, and unexpected metastases are discovered intraoperatively. In contrast, true M0 patients with benign pleural nodules and PE may be misdiagnosed as M1a on imaging alone and may lose the opportunity for radical resection. Therefore, accurate pleural staging is necessary to avoid futile surgical invasion in patients with pleural metastases that cannot be cured by radical resection and to potentially guide curable patients to appropriate surgical resection.

LAT is the gold standard procedure for diagnosing PE of unknown cause,^11^, ^12^ with a sensitivity of 91% and a specificity of 100%, as shown by a meta‐analysis of 17 studies.^13^ It may also be useful and safe for DPD.^7^, ^14^ Although previous studies have evaluated the diagnostic yield of LAT in patients with pleural metastases, there have been no data focusing on its potential role in pleural staging, especially in DPD and minimal PE.

This study aimed to explore the utility, applicability, and safety of LAT in the diagnosis of the early stage of pleural metastases.

## METHODS

### Study design and participants

This single‐center retrospective study included newly diagnosed patients with NSCLC who underwent LAT for pleural staging because of suspected DPD or minimal PE on CT and ^18^F‐fluorodeoxyglucose positron emission tomographic (^18^F‐FDG PET) scans from April 2015 to September 2020 at the National Cancer Center Hospital, Tokyo, Japan. This study was approved by the National Cancer Center Institutional Review Board (No. 2018‐090), and written informed consent for the LAT procedure was obtained from all study participants.

### Radiological assessment

CT scans with 1.0 to 5.0 mm collimation from the supraclavicular region to the diaphragm were obtained at least 1 month before the procedure in all cases. Both the standard mediastinal window and lung window were viewed. FDG PET/CT was also performed.

DPD and minimal PE were radiologically defined as “multiple small pleural nodules and/or uneven pleural thickenings without PE,”^5^, ^6^, ^7^, ^8^, ^9^ and “PE within 10 mm thickness that is too little to be punctured, with or without pleural lesions,”^10^ respectively.

### Procedures

**FIGURE 1:**
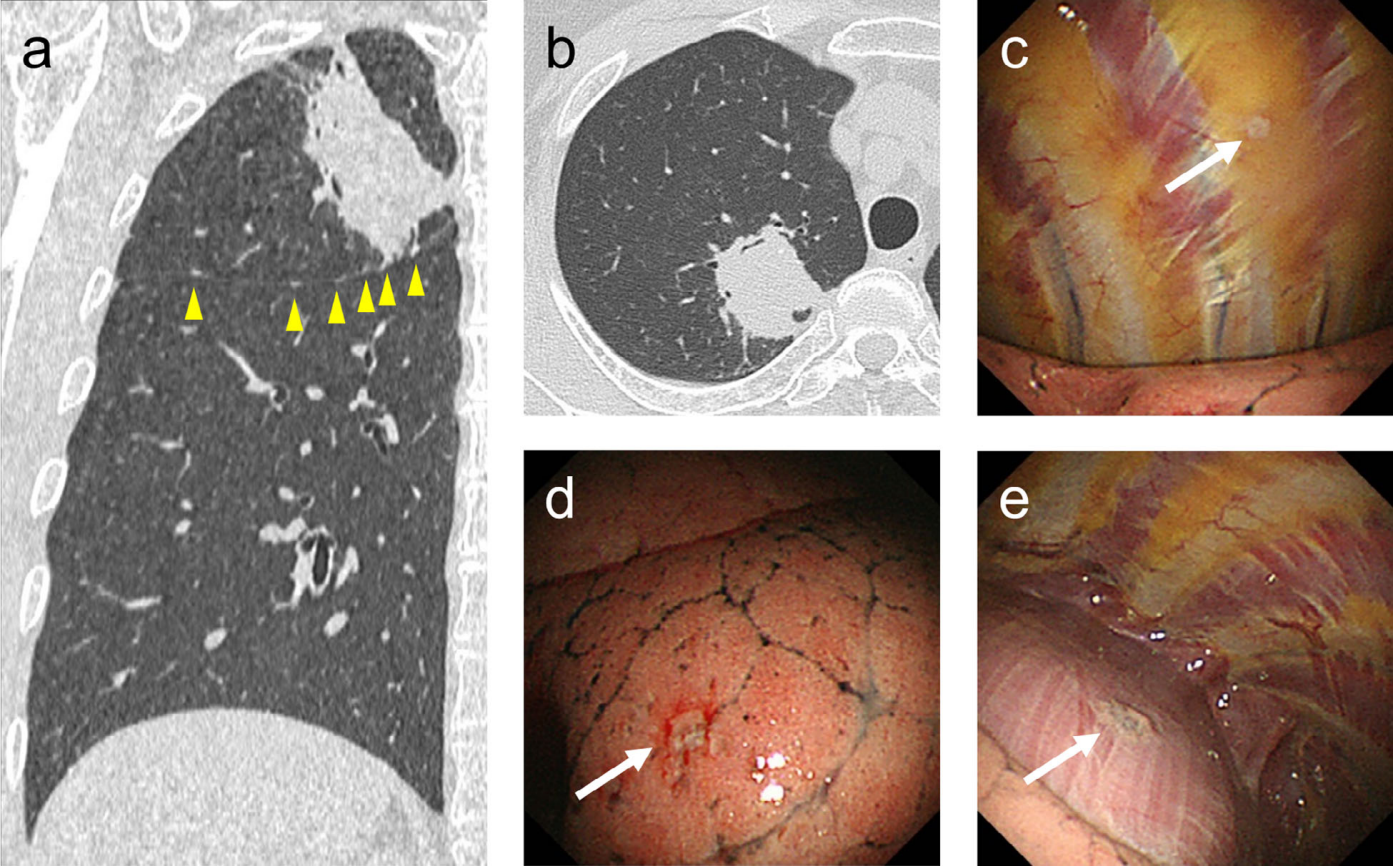
A case of dry pleural dissemination in a 69‐year‐old woman (case 4 in Table 2); (**a**) coronal and (**b**) axial sections of a computed tomographic scan (lung window) show a primary lesion abutting the right major fissure in the right upper lobe, pleural nodules only on the fissure (arrow head), and no pleural effusion. Local anesthetic thoracoscopic image reveals a small number of tiny, flat, and glossy nodules (arrow) without pleural effusion on the (**c**) parietal, (**d**) visceral, and (**e**) diaphragmatic pleura

All procedures were performed under local anesthesia in the endoscopy suite using a single‐puncture technique with a flex‐rigid pleuroscope (LTF‐260, Olympus, Tokyo, Japan). The patient was placed in the lateral decubitus position. The thoracic entry site was then, identified with transthoracic ultrasonography through the visualization of lung sliding. A local anesthetic (a mixture of 1% lidocaine and 1:100 000 adrenaline) to the chest wall and intravenous pentazocine was administered beforehand. Blunt dissection, trocar (MAJ‐1058, Olympus, Tokyo, Japan) placement, pleuroscope introduction, and thorough observation of the entire chest cavity, including the parietal, visceral, and diaphragmatic pleura (Fig 1), were performed. Abnormalities such as pleural thickening, pleural nodules, and adhesions were observed. Sedation with intravenous midazolam or propofol was performed when the patient complained of chest pain caused by the procedure. PE, if any, was collected for examination.

Pleural lesions suspected to be malignant were biopsied using the “lift and peel” technique with a local injection needle (NM‐9 L‐1 needle, Olympus, Tokyo, Japan) and standard flexible forceps (FB‐211D and FB‐210 K, Olympus, Tokyo, Japan), as described previously.^7^, ^14^ A cryoprobe with 2.4‐mm diameter (20402–032, Erbe Elektromedizin GmbH, Tübingen, Germany) was used when it was difficult to biopsy with forceps. The collected tissue samples were fixed in formalin and sent for histopathological analysis. A 20‐Fr chest tube was placed at the end of the procedure, and a chest radiograph was obtained. Any related complications were recorded.

### Data collection and analyses

The following variables were extracted from the electronic medical records and imaging database: age, sex, histological type, tumor location and its locational relationship with the pleura and fissure, clinical stage based on the 8th edition of the TNM classification, presence of minimal PE, presence and distribution of pleural/fissural thickenings and/or nodules, FDG uptake in these pleural lesions, epidermal growth factor receptor mutation or anaplastic lymphoma kinase fusion status, complications, procedure time from insertion of the pleuroscope into the chest cavity to the end of pleural biopsy, and results of the pleural staging and pathology.

All malignant diagnoses were confirmed pathologically. The benign diagnoses were established based on the histological results of a subsequent surgical resection. Pleural staging was deemed successful if the pathology results of LAT were consistent with the final diagnosis. Sensitivity, specificity, positive predictive value (PPV), and negative predictive value (NPV) were calculated using the final diagnosis as the gold standard by which the LAT diagnostic accuracy was tested. All data were statistically described in terms of frequencies (number of cases), percentages, medians, and ranges when appropriate.

## RESULTS

A total of 18 patients were included in the analysis. The baseline characteristics are described in Table 1. The median age of the patients was 72 years (range: 39–85 years), and nine of them (50.0%) were men. In all cases except three, the pathological diagnosis was confirmed by initial bronchoscopic biopsy, and LAT procedures were performed only for staging. The dominant histological type was adenocarcinoma (*n* = 16, 88.8%), with 12 cases (66.6%) showing driver mutations.

**TABLE 1:** The baseline characteristics of patients

Characteristics	
Median age (range) [years]	72 (39–85)
Sex	
Male	9 (50.0)
Female	9 (50.0)
Smoking history	
Never	10 (55.5)
Past	8 (44.4)
Clinical T factor^a^	
1	4 (22.2)
2	9 (50.0)
3	3 (16.6)
4	2 (11.1)
Clinical N factor^a^	
0	8 (44.4)
1	6 (33.3)
2	4 (22.2)
Primary lesion abutting on the pleura/fissure	17 (94.4)
Minimal pleural effusion	5 (27.7)
Fissural nodules	17 (94.4)
Pleural nodules	14 (77.7)
Pleural thickenings	8 (44.4)
Pleural lesions with FDG uptake	8 (44.4)
Histological type	
Adenocarcinoma	16 (88.8)
Squamous cell carcinoma	2 (11.1)
Driver mutation	
*EGFR* mutation	10 (55.5)
*ALK* fusion	2 (11.1)

Data are presented as N (%) unless otherwise noted.

^a^ The stage of all patients was defined according to the eighth edition of the TNM classification.

ALK, anaplastic lymphoma kinase; EGFR, epidermal growth factor receptor; FDG, fluorodeoxyglucose.

The number of cases in which the primary lesion was adjacent to the pleura or fissure, cases with fissural nodules, pleural nodules, and thickenings on CT scans were 17 (94.4%), 17 (94.4%), 14 (77.7%), and 8 (44.4%), respectively. FDG uptake of these pleural lesions was detected in eight cases (44.4%).

Table 2 outlines the results of LAT. Of the five patients with radiologically minimal PE, only three had visible PE on LAT images, and one of them was cytologically diagnosed as benign. Conversely, of the 13 patients with radiological DPD, three had a very small amount of PE (4–15 mL) collected by LAT, and cytology revealed malignant cells in two of them.

**TABLE 2:** Radiological and thoracoscopic findings of patients

Case no.	Primary site	Clinical T/N factor^a^	PE on CT	PE collected by LAT	FDG uptake of pleural lesions	Pleural nodules/thickenings on LAT images			Forceps biopsy	Cryobiopsy	Final diagnosis		Staging results
						Parietal	Diaphragmatic	Visceral			Histology	M factor^a^	
1	RUL	4/1	–	–	Present	Nodules	Nodules	Nodules	Malignant	–	AD	1a	Success
2	LLL	3/2	Minimal	Malignant	Present	Both	–	Nodules	Malignant	–	AD	1a	Success
3	RUL	2b/1	Minimal	–	Present	Both	Nodules	Nodules	Malignant	–	AD	1a	Success
4	RUL	2a/2	–	–	Present	Nodules	Nodules	Nodules	Malignant	–	AD	1a	Success
5	LLL	2a/0	–	–	–	Nodules	–	Nodules	Malignant	Malignant	AD	1a	Success
6	LUL	2a/2	–	–	–	Both	Nodules	Nodules	Malignant	Malignant	AD	1a	Success
7	LLL	1c/0	–	–	–	Nodules	Nodules	Nodules	Malignant	–	AD	1a	Success
8	RUL	3/1	–	–	–	–	–	–	–	–	AD	0	Success
9	LLL	2a/2	–	Benign	–	Nodules	–	–	Benign	–	AD	0	Success
10	LLL	4/1	–	–	Present	–	–	–	Benign^b^	–	SQ	1a	Failure
11	RUL	1c/0	–	Malignant	–	Both	Nodules	Nodules	Malignant	–	AD	1a	Success
12	RLL	3/0	Minimal	Malignant	–	Both	–	Nodules	Malignant	Malignant	AD	1a	Success
13	LLL	2a/0	–	Malignant	–	Nodules	Nodules	Both	Malignant	–	AD	1a	Success
14	RML	2b/0	–	–	Present	Nodules	–	Nodules	Malignant	–	AD	1a	Success
15	RLL	2a/1	Minimal	Benign	–	–	–	–	–	–	SQ	0	Success
16	RUL	1b/0	Minimal	–	Present	Nodules	Nodules	Nodules	Malignant	–	AD	1a	Success
17	RUL	1a/0	–	–	Present	Both	–	Nodules	Benign	Benign	AD	0	Success
18	RLL	2a/1	–	–	–	Both	Nodules	–	Malignant	Malignant	AD	1a	Success

^a^ The stage of all patients was defined according to the eighth edition of the TNM classification.

^b^ The site of pleural adhesions near the primary lesion was biopsied.

AD, adenocarcinoma; CT, computed tomography; FDG, fluorodeoxyglucose; LAT, local anesthetic thoracoscopy; LLL, left lower lobe; LUL, left upper lobe; PE, pleural effusion; RLL, right lower lobe; RML, right middle lobe; RUL, right upper lobe; SQ, squamous cell carcinoma.

**FIGURE 2:**
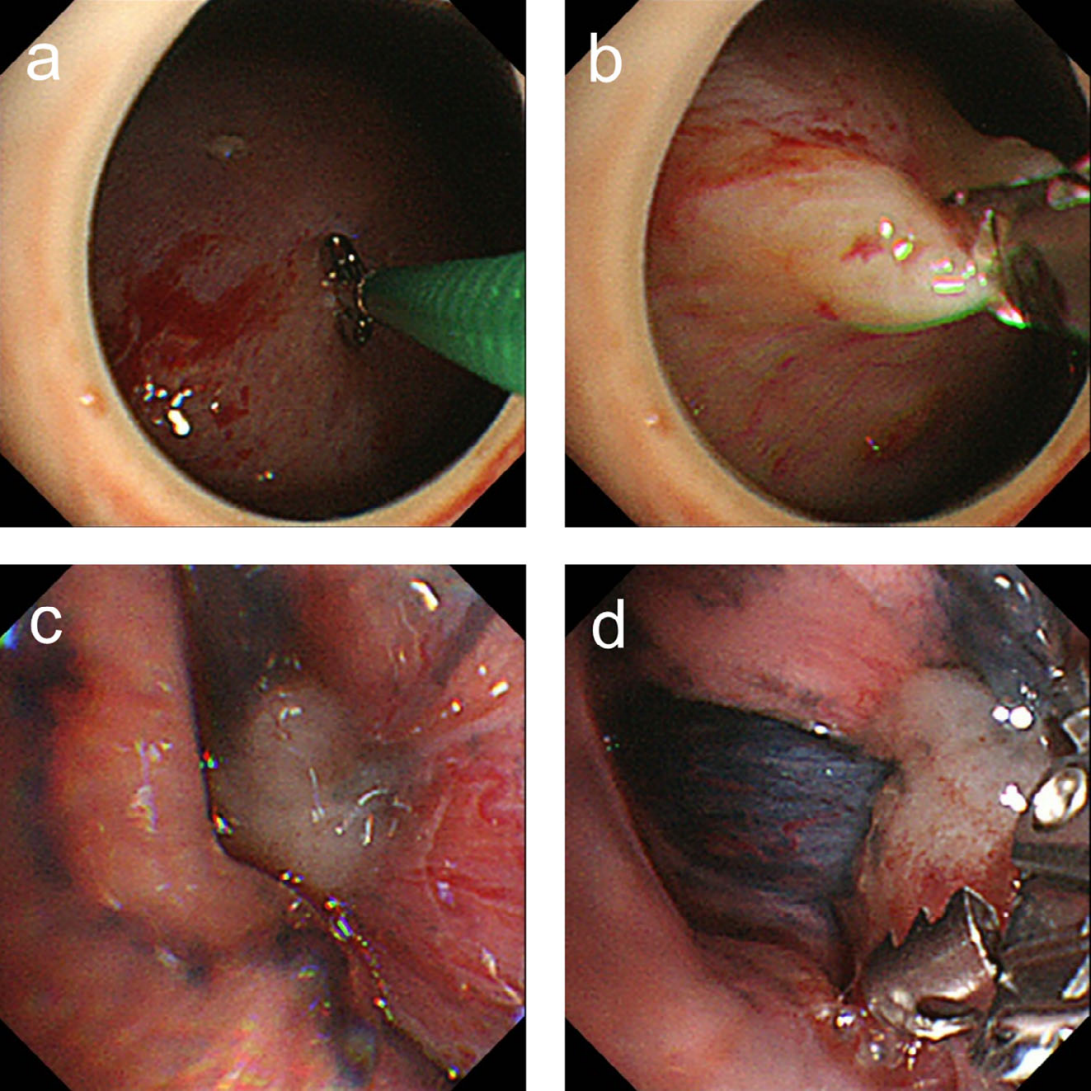
In some cases, local anesthetic thoracoscopic images did not show any obvious lesions highly suspective of malignancy on the parietal pleura, (**a**), (**b**) the diaphragmatic or (**c**), (**d**) the visceral pleural nodules were biopsied. These biopsy procedures are often difficult because of inaccessibility near the lesion or because of diaphragmatic stretching when biting off by forceps in the former and respiratory variation in the latter

**FIGURE 3:**
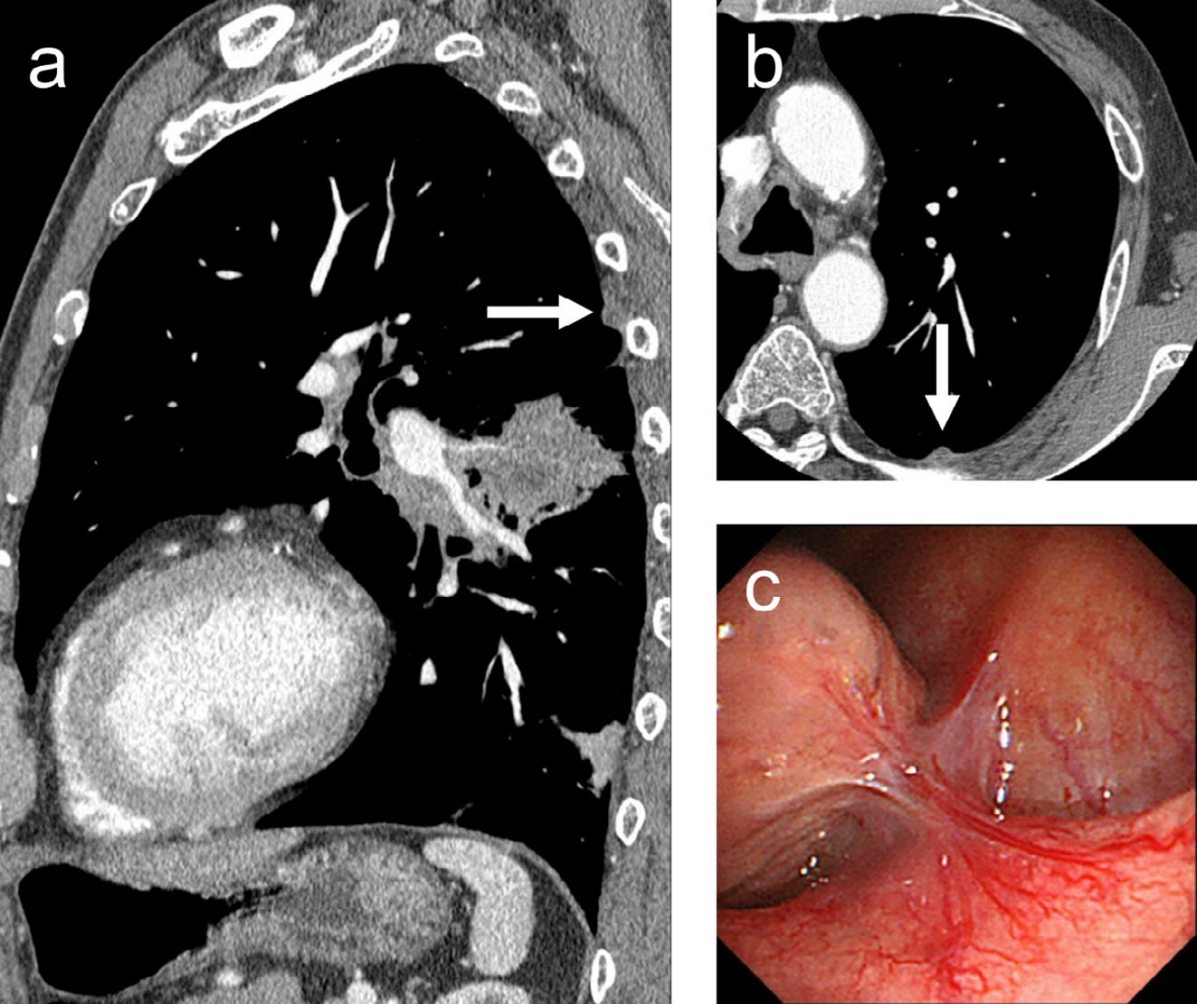
A case of failed pleural staging in a 78‐year‐old man (case 10 in Table 2). (**a**) Sagittal and (**b**) axial sections of a computed tomographic scan (mediastinal window) show a pleural nodule (arrow) on the head side of the primary lesion in the left lower lobe. (**c**) this nodule could not be identified on local anesthetic thoracoscopy because of the adhesion between the chest wall and lung

Pleural biopsy was performed in all except two cases in which no obvious pleural lesion suspected of malignancy was observed. The sampling devices used were forceps in all cases and cryoprobes in five cases. The biopsy sites were the parietal pleura in 14 cases, the diaphragmatic pleura in three cases, the visceral pleura in two cases (Fig 2), and pleural adhesions in one case (Fig 3; case 10 in Table 2). Pleural biopsy confirmed pleural dissemination in 13 cases. Four of the other five patients underwent subsequent surgical resection and were diagnosed with pathological M0 (true–negative). The one remaining case was deemed unresectable because of the involvement of the descending aorta associated with left back pain (clinical T4). It was clinically diagnosed as M1a (false–negative) with the appearance of malignant PE 5 weeks after LAT, confirmed by thoracentesis 3 weeks later during follow up LAT (case 10 in Table 2). Hence, the success rates for pleural staging, sensitivity, specificity, PPV, and NPV were 94.4% (17/18), 92.8% (13/14), 100% (4/4), 100% (13/13), and 80.0% (4/5), respectively.

The median procedure time was 21.3 (range: 13.5–86.0) min. Mild chest pain occurred in six patients, and agitation/delirium was observed in two patients during examination. There were no lung lacerations or other severe complications caused by the procedure or during blunt dissection.

## DISCUSSION

The current study demonstrated that pleural staging using LAT safely provided diagnostic accuracy in 94.4% of NSCLC patients with radiologically suspected DPD or minimal PE. As a result of this staging, 14 patients diagnosed with M1a were able to avoid futile surgical invasion and initiate drug therapy based on their respective histological type, gene mutation/fusion status, and expression of programmed death‐ligand 1. Three of the four patients diagnosed with M0 were able to undergo radical surgical resection.

Previous studies have described that DPD and minimal PE indicate an early phase of pleural metastases and are important prognostic factors. Because it takes an average of 19 to 41.9 months for the development of malignant PE from DPD,^6^, ^9^ patients with DPD survive much longer than patients with malignant PE (median survival of 38 vs. 13 months, *P* < 0.001).^6^ Similarly, patients with minimal PE have poorer prognosis than those without PE.^10^, ^15^ However, the following limitations have been mentioned; (a) only pathologically proven DPD cases were included, and clinically diagnosed DPD were excluded from the analysis,^6^ and (b) a definite diagnostic procedure such as pleural biopsy was not attempted in the majority of minimal PE cases because it was either technically unfeasible or a change in treatment strategy was not anticipated.^15^ Therefore, the extended use of LAT may help to solve some of these problems and determine the prognostic impact of DPD and minimal PE.

In determining the indication for pleural staging using LAT, CT scans are the most important diagnostic work‐up to help detect DPD and minimal PE. Previous studies have revealed that DPD can appear more frequently when the primary lesion is adjacent to the pleura or the fissure,^16^, ^17^ when there are multiple small pleural nodules and uneven or band‐like fissural thickenings.^5^ Furthermore, CT scans have been shown to have higher sensitivity and specificity when integrated with PET.^5^ If PET/CT findings are obviously positive for malignancy, LAT may be avoided considering its invasiveness. However, PET/CT and LAT findings do not always match. In the present study, some radiologically silent pleural lesions were identified on LAT images. Conversely, some radiologically observed pleural lesions could not be identified on LAT images because of pleural adhesions or poor lung collapse.^14^

Moreover, some pleural abnormalities were not malignant. It has been reported that 17% to 18% of the analyzed PE in patients with lung cancer were unrelated to pleural malignancy (e.g., post‐surgery, pneumonia, or heart failure).^15^, ^18^ In fact, 33.3% (2/6) of minimal PE in the present study were also benign fluids. According to the diagnostic criteria for DPD,^8^ less than six pleural/fissural nodules or thickenings may represent histologically benign lesions (e.g., intrapulmonary lymph nodes, anthracofibrotic nodules, or granulomas). False‐positive PET scans may be seen as a result of fractured ribs, artifacts due to poor resolution, or nodular atelectasis.^5^, ^19^ Therefore, if technically feasible, histological confirmation by LAT can be useful to reduce misjudgments of the surgical indications.

In cases with more localized pleural seeding, it is essential to seek out and biopsy as many lesions as possible on LAT images for a definite diagnosis. Regardless of the location of the primary lesion, the area around the costophrenic angle, where pleural abnormalities are often distributed, should be carefully explored. The diaphragmatic or visceral pleura, as well as the parietal pleura, may be subject to biopsy. In particular, visceral pleural biopsy as well as LAT in patients with minimal or no PE is listed as a level II technique that should be performed by more experienced practitioners with a major interest in pleural diseases.^11^ Although some argue that visceral pleural biopsy should be avoided because of the risk of prolonged air leak,^20^ there has been a case report of safe and successful diagnosis.^21^

With regard to the position of LAT in the diagnostic pathway, medical thoracoscopists should fully discuss the balance of risks and benefits in individual patients and whether LAT or surgical procedures such as video‐assisted thoracoscopic surgery and thoracotomy are the optimal treatment strategy.^11^ Compared with surgical procedures, the proposed advantages of LAT include a smaller incision site and lower anesthetic requirements. LAT allows for pleural exploration and biopsies with safety even in patients with minimal/no PE, with the aid of transthoracic ultrasonography to visualize lung sliding and most importantly, avoid pleural adhesions. Conversely, the disadvantages of LAT include a limited field of view in the thoracic cavity because of the single‐puncture technique, especially in cases with extensive pleural adhesions or poor lung collapse and in smaller specimens obtained with forceps. Cryobiopsy is considered easy and quick to handle even in such situations,^14^, ^22^ and it is expected that a sampling technique that allows for larger and better quality specimens will have a sufficient sample for next‐generation sequencing.^23^, ^24^, ^25^, ^26^

Our study had some limitations. First, it was conducted at a single cancer center, leading to possible bias in patient selection. Second, it was a retrospective analysis with a small sample size. Third, the present study did not investigate the prognosis of patients who underwent pleural staging using LAT and did not compare them with those who underwent surgical procedures, for the following reasons: (a) pleural staging was not performed in all patients with DPD and minimal PE suspected radiologically, and (b) in some cases, prognostic analysis was not available because of the short time course after pleural staging. Although still controversial, due in part to recent advances in systemic therapy, a meta‐analysis and nine retrospective studies indicated that resection of a primary tumor was a beneficial prognostic factor among NSCLC patients with pleural metastases detected unexpectedly during surgery.^27^, ^28^, ^29^, ^30^ Future studies are required to determine whether pleural staging would contribute to improving the prognosis of these patients. Although the rarity of these patients is certainly a concern, larger, prospective, randomized controlled trials are needed to validate these findings. Despite these limitations, our study validates the technical feasibility of performing LAT for cases of DPD and minimal PE.

In conclusion, LAT might be a useful tool for accurate pleural staging in cases with DPD and minimal PE suspected radiologically.

## DISCLOSURE

The authors declare no conflicts of interest.
